# Supplementary material for: Modeling glioblastoma heterogeneity as a dynamic network of cell states
Source: Mol Syst Biol. 2021 Sep 16;17(9):e10105. doi: 10.15252/msb.202010105 (PMC8444284; doi:10.15252/msb.202010105)
Supplement: Supplementary file 5 — Source Data for Figure 3 [file MSB-17-e10105-s001.zip › Figure3A_sourcedata/GSEA_3065/nosigdb_state4.GseaPreranked.1622718430994/NB_KI_CLUSTER4.html]

Details for gene set NB\_KI\_CLUSTER4[GSEA]

|  || Dataset | state4 |
| Phenotype | NoPhenotypeAvailable |
| Upregulated in class | na\_pos |
| GeneSet | NB\_KI\_CLUSTER4 |
| Enrichment Score (ES) | 0.48014215 |
| Normalized Enrichment Score (NES) | 1.955055 |
| Nominal p-value | 0.0 |
| FDR q-value | 0.0022091088 |
| FWER p-Value | 0.017 |
Table: GSEA Results Summary

  

Fig 1: Enrichment plot: NB\_KI\_CLUSTER4      
 Profile of the Running ES Score & Positions of GeneSet Members on the Rank Ordered List

  

| PROBE | GENE SYMBOL | GENE\_TITLE | RANK IN GENE LIST | RANK METRIC SCORE | RUNNING ES | CORE ENRICHMENT || 1 | TUBA1A |  |  | 15 | 0.460 | 0.0369 | Yes |
| 2 | NLRP1 |  |  | 16 | 0.425 | 0.0733 | Yes |
| 3 | SOX4 |  |  | 18 | 0.402 | 0.1076 | Yes |
| 4 | TUBB2B |  |  | 24 | 0.373 | 0.1387 | Yes |
| 5 | PTMS |  |  | 25 | 0.372 | 0.1706 | Yes |
| 6 | BEX3 |  |  | 35 | 0.337 | 0.1980 | Yes |
| 7 | MARCKSL1 |  |  | 52 | 0.295 | 0.2206 | Yes |
| 8 | TUBB2A |  |  | 61 | 0.287 | 0.2439 | Yes |
| 9 | TTC3 |  |  | 85 | 0.261 | 0.2625 | Yes |
| 10 | SNRPN |  |  | 101 | 0.247 | 0.2812 | Yes |
| 11 | APLP1 |  |  | 102 | 0.246 | 0.3024 | Yes |
| 12 | MAP1B |  |  | 111 | 0.243 | 0.3219 | Yes |
| 13 | GTF2I |  |  | 128 | 0.236 | 0.3396 | Yes |
| 14 | H3F3A |  |  | 189 | 0.207 | 0.3476 | Yes |
| 15 | MYL6B |  |  | 210 | 0.201 | 0.3616 | Yes |
| 16 | UCHL1 |  |  | 232 | 0.190 | 0.3744 | Yes |
| 17 | MLLT11 |  |  | 267 | 0.182 | 0.3845 | Yes |
| 18 | GNAS |  |  | 280 | 0.179 | 0.3979 | Yes |
| 19 | DPYSL3 |  |  | 298 | 0.174 | 0.4100 | Yes |
| 20 | TERF2IP |  |  | 386 | 0.157 | 0.4094 | Yes |
| 21 | FSCN1 |  |  | 428 | 0.147 | 0.4153 | Yes |
| 22 | H3F3B |  |  | 447 | 0.144 | 0.4248 | Yes |
| 23 | PEBP1 |  |  | 449 | 0.144 | 0.4369 | Yes |
| 24 | GDI1 |  |  | 467 | 0.141 | 0.4463 | Yes |
| 25 | RUFY3 |  |  | 567 | 0.129 | 0.4413 | Yes |
| 26 | TRIM2 |  |  | 580 | 0.128 | 0.4503 | Yes |
| 27 | KIF21A |  |  | 627 | 0.123 | 0.4534 | Yes |
| 28 | ATAT1 |  |  | 650 | 0.120 | 0.4601 | Yes |
| 29 | TCAF1 |  |  | 717 | 0.112 | 0.4590 | Yes |
| 30 | YWHAE |  |  | 724 | 0.111 | 0.4675 | Yes |
| 31 | MEG3 |  |  | 756 | 0.108 | 0.4718 | Yes |
| 32 | NREP |  |  | 810 | 0.103 | 0.4720 | Yes |
| 33 | MCRIP1 |  |  | 815 | 0.103 | 0.4801 | Yes |
| 34 | TMEM14A |  |  | 899 | 0.096 | 0.4749 | No |
| 35 | FXYD6 |  |  | 960 | 0.091 | 0.4730 | No |
| 36 | ATCAY |  |  | 1041 | 0.085 | 0.4673 | No |
| 37 | PRKAR2B |  |  | 1084 | 0.083 | 0.4676 | No |
| 38 | ICA1 |  |  | 1134 | 0.080 | 0.4665 | No |
| 39 | RBP1 |  |  | 1171 | 0.078 | 0.4673 | No |
| 40 | YWHAQ |  |  | 1251 | 0.073 | 0.4608 | No |
| 41 | QDPR |  |  | 1398 | 0.066 | 0.4429 | No |
| 42 | BEX2 |  |  | 1754 | 0.054 | 0.3900 | No |
| 43 | CKB |  |  | 1774 | 0.053 | 0.3915 | No |
| 44 | STXBP1 |  |  | 1917 | 0.049 | 0.3727 | No |
| 45 | CFAP20 |  |  | 1939 | 0.048 | 0.3734 | No |
| 46 | MTSS1 |  |  | 1988 | 0.047 | 0.3697 | No |
| 47 | CBX1 |  |  | 2104 | 0.044 | 0.3548 | No |
| 48 | KIF3A |  |  | 2116 | 0.043 | 0.3568 | No |
| 49 | CDK6 |  |  | 2322 | 0.039 | 0.3269 | No |
| 50 | SUMO2 |  |  | 2329 | 0.038 | 0.3292 | No |
| 51 | PEG10 |  |  | 2338 | 0.038 | 0.3312 | No |
| 52 | ATP6V0E2 |  |  | 2393 | 0.037 | 0.3256 | No |
| 53 | MYEF2 |  |  | 3097 | 0.023 | 0.2138 | No |
| 54 | RBFOX2 |  |  | 3386 | 0.016 | 0.1685 | No |
| 55 | HDAC2 |  |  | 3407 | 0.015 | 0.1666 | No |
| 56 | VAMP2 |  |  | 3479 | 0.013 | 0.1562 | No |
| 57 | CALM1 |  |  | 3619 | 0.008 | 0.1344 | No |
| 58 | COX6A1 |  |  | 3753 | 0.003 | 0.1131 | No |
| 59 | CADM1 |  |  | 3798 | 0.001 | 0.1060 | No |
| 60 | CALM2 |  |  | 3844 | -0.001 | 0.0988 | No |
| 61 | CIRBP |  |  | 3948 | -0.007 | 0.0827 | No |
| 62 | C3orf14 |  |  | 4079 | -0.014 | 0.0629 | No |
| 63 | ZC3H13 |  |  | 4154 | -0.019 | 0.0525 | No |
| 64 | GLCCI1 |  |  | 4365 | -0.033 | 0.0213 | No |
| 65 | NSD3 |  |  | 4418 | -0.037 | 0.0161 | No |
| 66 | MIAT |  |  | 4438 | -0.039 | 0.0163 | No |
| 67 | STMN3 |  |  | 4493 | -0.044 | 0.0114 | No |
| 68 | YWHAH |  |  | 4595 | -0.051 | -0.0006 | No |
| 69 | SYT11 |  |  | 4599 | -0.051 | 0.0033 | No |
| 70 | TSPAN13 |  |  | 4822 | -0.069 | -0.0268 | No |
| 71 | ZNF704 |  |  | 4861 | -0.072 | -0.0267 | No |
| 72 | OCIAD2 |  |  | 5014 | -0.084 | -0.0441 | No |
| 73 | NME1 |  |  | 5086 | -0.090 | -0.0479 | No |
| 74 | RIC3 |  |  | 5103 | -0.092 | -0.0426 | No |
| 75 | MDK |  |  | 5110 | -0.093 | -0.0357 | No |
| 76 | JPT1 |  |  | 5145 | -0.096 | -0.0329 | No |
| 77 | CXADR |  |  | 5248 | -0.105 | -0.0404 | No |
| 78 | ATP6V0A1 |  |  | 5273 | -0.108 | -0.0351 | No |
| 79 | PHF14 |  |  | 5468 | -0.127 | -0.0556 | No |
| 80 | TUBB |  |  | 5482 | -0.129 | -0.0466 | No |
| 81 | NELFCD |  |  | 5614 | -0.146 | -0.0553 | No |
| 82 | SEC11C |  |  | 5750 | -0.172 | -0.0624 | No |
| 83 | LUC7L3 |  |  | 5954 | -0.224 | -0.0761 | No |
| 84 | SLC38A1 |  |  | 5972 | -0.228 | -0.0593 | No |
| 85 | CENPV |  |  | 5984 | -0.232 | -0.0412 | No |
| 86 | STMN1 |  |  | 5993 | -0.236 | -0.0223 | No |
| 87 | HSP90AB1 |  |  | 6119 | -0.312 | -0.0157 | No |
| 88 | CCND1 |  |  | 6203 | -0.454 | 0.0097 | No |
Table: GSEA details [plain text format]

  

Fig 2: NB\_KI\_CLUSTER4: Random ES distribution      
 Gene set null distribution of ES for **NB\_KI\_CLUSTER4**
